# Optimized extraction and kinetic study of cholesterol oxidase from a newly isolated *Escherichia fergusonii* strain from local whey samples: insights through a combined experimental study and artificial neural network modeling

**DOI:** 10.1186/s12866-024-03728-0

**Published:** 2025-01-20

**Authors:** Simin Khataee, Gholamreza Dehghan, Samaneh Rashtbari, Arezu Marefat, Sina Jamei, Hamed Farzi-Khajeh

**Affiliations:** 1 Laboratory of Biochemistry and Molecular Biology, Department of Biology, Faculty of Natural Sciences, University of Tabriz, Tabriz, 51666−16471 Iran; 2 Liver and Gastrointestinal Diseases Research Center, Tabriz University of Medical Sciences, Tabriz, Iran; 3 Arg Biotech Company, East Azarbaijan Science and Technology Park, Tabriz, Iran

**Keywords:** *Escherichia fergusonii*, Cholesterol oxidase, 16S rRNA gene, Artificial neural network, Optimization

## Abstract

**Background:**

Microbial cholesterol oxidase (ChoX) has wide clinical and industrial applications; therefore, many efforts are being made to identify promising sources. This study aimed to isolate a novel ChoX-producing bacterial strain from whey samples.

**Results:**

The most efficient strain was selected based on extracellular ChoX-producing ability and characterized as *Escherichia fergusonii* (*E. fergusonii*) through molecular and biochemical analysis. The maximum production of ChoX was obtained at the optimum condition of 48 h of incubation under shaking conditions (130 rpm) at 35 °C in a basal medium adjusted to pH 6.5, including 1.4 g/L cholesterol as a sole carbon. The crude product was purified by ammonium sulfate precipitation and followed by ion exchange chromatography utilizing Q-Sepharose, resulting in 5.35-fold and 13.86-fold purification, respectively, with a final specific activity of 15.8 U/mg. Additionally, molecular weight was determined by SDS-PAGE to be 49.0 kDa. The optimum conditions required for the higher cholesterol decomposition ability of purified ChoX were suggested to be 30 °C and pH 7.5 in the presence of MgSo_4_ with a *K*_*m*_ value of 0.71 mM. However, other case studies of metal ions showed an unfavorable effect on enzymatic performance. The enzyme retained almost 72.0% of its initial activity after 80 days of storage at 4 °C. Furthermore, the ChoX enzyme revealed acceptable stability at a pH value of 6.5 to 8.5, maintaining its initial activity of more than 50.0%. Finally, an artificial neural network (ANN) was designed to predict the most effective factor in the fermentation process for enzyme production and the purified ChoX activity.

**Conclusions:**

Considering the properties of the extracted enzyme from *E. fergusonii*, it would be regarded as a potential ChoX source for commercial applications.

**Supplementary Information:**

The online version contains supplementary material available at 10.1186/s12866-024-03728-0.

## Background

Cholesterol oxidase (ChOx: EC 1.1.3.6), as a flavin adenine dinucleotide-dependent enzyme, catalyzes the oxidation and isomerization of cholesterol to form 4-cholesten-3-one and hydrogen peroxide in the presence of molecular oxygen. The role of cholesterol determination in evaluating health conditions such as atherosclerosis, coronary heart disease, thrombosis, and other lipid disorders has caused serious demand for ChOx, which is an essential element for the clinical detection of blood serum cholesterol [1]. Furthermore, ChOx is considered one of the most extensively utilized enzymes in pharmaceuticals and industrial laboratories due to its application potential for biotechnological purposes. Indeed, ChOx is a fundamental enzyme for the synthesis of steroid drugs [2], the evaluation and decomposition of dietary cholesterol in foods by coupling the enzyme with peroxidase [3], and its usage as a biological insecticidal in crop pest management [4]. ChOx can also be used for the production of enzymatic biosensors and diagnostic kits to determine the cholesterol level in various samples [5]. Bacteria utilize ChOx for the breakdown of cholesterol in host macrophages to benefit the carbon source [6]. Most microbial sources produce intracellular and membrane-bound ChOx enzyme; however, bacterial strains producing extracellular forms are of great importance due to the more accessible screening and isolation of the enzyme.

On the other hand, the genus *Escherichia* belongs to the family of Enterobacteriaceae and is composed of gram-negative, non-spore-forming, facultative aerobes, and rod-shaped bacteria. Escherichia is easily cultured in the clinical laboratory with controllable fermentation conditions. The first identified species, *Escherichia coli* (*E. coli*), has become by far the most investigated bacterium in the microbiological field [22]. Afterward, other species were recognized in this genus, including *Escherichia fergusonii* (*E. fergusonii*) as the closest relative of E. coli with the most similarity in DNA sequencing [23]. *E. fergusonii* is a rare opportunistic pathogen in humans and animals that causes diseases only infrequently [24]. It is isolated from diverse natural environments, clinical, and non-clinical sources, such as wound infections [7], diarrhea [8], water [23], and dairy products [24]. As far as we know, there are not any published articles based on the ChOx extraction from *E. fergusonii*; however, some articles have reported the potential of this bacteria for the extracellular production of chitinase [9].

Since ChOx, as a versatile enzyme, has many applications in food and medical fields, the identification of efficient enzyme-producing organisms is of vital importance [10]. Accordingly, investigation has proceeded to identify novel microorganisms capable of generating such an industrially significant enzyme with appropriate properties. In the present work, *E. fergusonii* was introduced as a new microbial source of ChOx. The most active isolates of cholesterol-decomposing bacteria were isolated from dairy products (whey) and identified at the molecular level by sequencing the 16S rRNA gene region. The biochemical properties of the bacteria were investigated. The kinetic properties and activity of the ChOx are considerably affected by the environment of its substrate [11]. Therefore, the optimization of culture conditions has a significant role when studying cholesterol oxidase enzymes in bacteria to enhance microbial growth and increase the productivity of enzyme extraction. Therefore, medium optimization and fermentation studies were applied by evaluating the effects of nutrients, temperature, and pH conditions to suggest the efficient elements that influence the production of cholesterol-decomposing enzymes. Ion exchange chromatography and SDS-PAGE electrophoresis methods were performed for the enzyme’s purification and molecular weight determination, respectively. Subsequently, the produced enzyme was subjected to a study of the effects of substrate affinity, thermal property, pH tolerance, and metal ions on enzyme activity.

## Methods

### Materials and media components

All chemicals and bacterial media ingredients were purchased from Sigma Aldrich (St. Louis, MO, USA) and used without further purification. The compositions of PCR, including master mix and primers, were received from Gen All (Korea) and Bioneer (Korea) companies, respectively. Protein molecular weight markers and DNA ladders were brought from Sinaclon (Iran). The gel filtration column and Q-sepharose were obtained from Arg Biotech Company (Iran).

### Isolation of cholesterol-decomposing microorganisms

A total of 15 local whey samples were collected from various dairy product factories in Tabriz, Iran, to isolate ChOx-producing microorganisms. Each 1.0 mL sample was suspended in 100 mL of a 0.9% NaCl solution and shaken for 30 min. Single bacterial colonies were then isolated using the four-zone cultivation technique on Luria–Bertani (LB) broth medium (1.5% agar, 1.0% tryptone, 0.5% yeast extract, and 0.5% NaCl) [3]. Then, to investigate the cholesterol decomposition properties, each single colony was cultured on a cholesterol-enriched agar plate. The medium consisted of 15.0 g agar and 1.0 g cholesterol (as the sole carbon source) dissolved in 1.0 L of 0.5% Triton X-100. The pH of all media was adjusted to 7.0 (microprocessor pH meter; HANA Instruments, USA) before sterilization at 0.5 atm, associated with a temperature of 120 °C for 30 min (autoclave; RT-2 FW, Reyhan TB, Iran). After incubating the plates at 37°C for 3–6 days, bacterial colonies were seeded on the surface of agar plates [11].

The cholesterol-decomposing potential was confirmed by subculturing each colony on a ChOx indicator plate containing 0.5% cholesterol, 6.0% phenol, 1.7% 4-aminoantipyrine (4-AMAP), 1.5% agar, and 3000 U/L horseradish peroxidase (HRP) in 100 mM potassium phosphate buffer (KPB; pH 7.0). To ensure accurate and comparable results, an equal bacterial concentration of 0.5 × 10^6^ CFU/mL was used [12, 13]. After incubating at 37 °C for 4 days, the cholesterol oxidation ability of selected colonies was assessed by monitoring the time-required appearance of a red color. This red color indicates the presence of quinoneimine dye, which is a product of the cholesterol oxidation reaction [14]. Colonies that developed a deep red color, indicating higher cholesterol oxidase production, were selected for further screening tests and identification.

### Quantitative screening of ChOx producer colonies

The extracellular enzymatic activity was measured to identify the isolate with the highest cholesterol oxidation property in the broth medium. Each selected isolate, prepared as a 1.0% bacterial suspension, was cultured in 5.0 mL sterile test tubes containing cholesterol-enriched medium (1.0 g/L). The cultures were incubated for 16 h at 37 °C with continuous shaking at 200 rpm. Then, the cultures were centrifuged at 6000 rpm for 15 min at 4 °C to collect the bacterial cells, and the supernatant was used as the crude enzyme solution. The activity of extracellular ChOx was measured spectrophotometrically (T60, PG Instruments Ltd., Leicestershire, UK) using the method described by Sasaki et al. [15]. In this method, hydrogen peroxide (H_2_O_2_), generated from cholesterol oxidation, reacts with 4-AMAP and phenol in the presence of HRP to produce a quinoneimine dye with maximum absorption at 500 nm. For this purpose, the reaction mixture, including 50.0 μL of cholesterol 0.1 mM (2.5 µM) (dissolved in 1.0% Triton X-100), 0.2 mg/mL of the obtained ChOx solution, 1.0 µM 4-AMAP, 15.0 µM of phenol, and 10.0 U/mL of HRP in 0.1 M KPB (pH 7.0), was incubated at 37 °C for 2 min. One unit (U) of the enzyme was defined as the amount of ChOx that produces 1.0 μmol of the product (H_2_O_2_ as the by-product and 4-cholesten-3-one as the main product) per minute at 37 °C. The isolate with the best enzymatic activity, which was considered the most efficient enzyme-producing microorganism, was identified through conventional microbiological analyses [16].

### Identification and characterization of ChOx-producing isolate

The target strain was identified through morphological, biochemical, and molecular methods. The molecular identification was carried out through 16S rRNA sequencing and phylogenetic analysis. To achieve this, the strain was cultured in LB medium, and then chromosomal DNA was extracted as follows: Freshly grown bacteria were transferred to 1.0 mL KPB (0.01 M; PH 7.0) and centrifuged at 7000 rpm for 20 min [17]. The resulting precipitation was dissolved in 200 μL Tris–EDTA (TE) buffer (1.0 M; pH 7.5), heated at 95°C for 10 min, and then centrifuged at 9000 rpm for 20 min to collect the soluble DNA.

Afterward, PCR amplification of the 16S rRNA gene was accomplished using a couple of the universal bacterial primers (Forward 27: 5'-AGAGTTTGA TCMTGGCTC-3' and Reverse 1492: 5'-TACGGYTACCTTGTTACGACT-3'). The PCR reaction mixture consisted of 1.5 µL DNA template, 12.5 µL master mix, 1.0 µL of each primer, and 9 µL of double distilled water, making a final volume of 25.0 µL. The reaction began with an initial denaturation at 94 °C for 10 min, followed by 35 cycles of denaturation at 94 °C for 1 min, annealing at 54 °C for 1 min, and extension at 72 °C for 2 min. A final extension step was performed at 72 °C for 10 min [18]. Eventually, the PCR product was analyzed on 0.8% (w/v) agarose gels stained with ethidium bromide using electrophoresis. The PCR product was then purified utilizing a gel extraction kit (Bioneer, South Korea) and sequenced by Bioneer Co. (South Korea). The resulting sequence was compared with the nucleotide databases found on the National Center for Biotechnology Information (NCBI) website (http://www.ncbi.nlm.nih.gov) using the BLAST algorithm [19].

Additional studies were conducted to confirm the morphology and gram characteristics of bacteria with optical and scanning electron microscopes (SEM). To investigate the morphological structure, the bacterial strain was grown in LB broth medium for 36 h at 37 °C and then centrifuged and washed with 0.01 M PBS buffer. The obtained bacteria precipitate was fixed with a 2.5% glutaraldehyde solution at 37 °C for 12 h. Subsequently, the precipitation was subjected to different dilutions of ethanol solutions (10.0–100%) for dehydration and was studied with an SEM microscope [20]. The fundamental steps of the gram staining procedure were performed by applying crystal violet as a primary stain, Gram’s iodine as a mordant, decolorization phases with ethanol, acetone, or a mixture of both, and safranin as a final stain.

Furthermore, the antibiogram test was performed to determine the resistance pattern of the identified strain against various antibiotics, including vancomycin (VAN), cefazolin (CFZ), ceftriaxone (CTX), penicillin (PCN), ciprofloxacin (CIP), amikacin (AMK), amoxicillin (AMX), and levofloxacin (LEV), through a disc diffusion assay. Hence, the paper disks with a specific concentration of antibiotics are placed on the newly lawn-cultured bacteria in the LB medium. After incubation for a certain period, the diameter of the zone of inhibition is measured, and the results are reported as the percentage of resistance to the antibiotics.

The number of other oxidases secreted by *E. fergusonii* was evaluated using a 10.0% native polyacrylamide gel electrophoresis (PAGE) [21]. For this purpose, bacteria were cultured in LB medium containing 0.1% cholesterol. After incubating for 24 h at 37 °C (200 rpm), cultures were centrifuged at 6000 rpm for 15 min at 4 °C. The supernatant was utilized as the crude enzyme solution. A volume of 20.0 µL of sample with two different dilutions (the crude enzyme extract and its 1:4 dilution). was loaded on the native gels and run for 3 h. The zymogram patterns were visualized by an enzyme-specific staining approach. Accordingly, after incubation in distilled water for 2 h at 4°C, the gels were immersed in different reaction solutions containing different enzyme substrates. The possibility of peroxidase, glucose oxidase, and laccase secretion was monitored using 4.0 mM 3,3′,5,5′-tetramethylbenzidine (TMB) in 0.2 M sodium acetate buffer (pH 4.8) [22]. However, hydrogen peroxide (3.0% v/v) was also added to the peroxidase reaction solution. As well, the glucose oxidase reaction mixture contained 50.0 mM glucose and 3000 U/L HRP. The cholesterol oxidase (ChOx) reaction solution contained the same components used for enzyme activity analysis [23]. The gel electrophoresis images were captured using a smartphone camera and subsequently adjusted to 300 DPI resolution in Photoshop CS6 Portable, ensuring that no excessive contrast was applied.

To optimize ChOx production in the target strain, the effects of different physical parameters were systematically examined in broth medium. These parameters included incubation time (12–132 h), temperature (15 to 65 °C), pH levels (pH 5.0 to 10.0), and shaking conditions (50–250 rpm). The study also evaluated different concentrations of the substrate (0.05 to 2.0 g/L) and tested the influence of various metal salts (0.5 g/L), such as (NH_4_)_2_SO_4_, K_2_HPO_4_, MgSO_4_, and NaCl, to identify optimal conditions for enzyme activity. To ensure precise measurements, each experiment was repeated three times [17]. The optimized conditions were applied to produce the enzyme for further analysis.

### Purification of isolated ChOx from *E. fergusonii*

The cultures were centrifuged at 6000 rpm for 15 min at 4 °C to collect the bacterial cells, and the supernatant was used as the crude enzyme solution. To partially purify the enzyme, the crude enzyme was treated with 60.0% ammonium sulfate at 4 °C overnight. The proteins were then collected by centrifugation at 8,000 rpm for 30 min at 4 °C, and the supernatant was further saturated up to 80.0% with ammonium sulfate. In the next step, the precipitate was gathered through centrifugation and resuspended in 0.02 M KPB (pH 7.0). The obtained solution was dialyzed against the same buffer at 4 °C overnight with a dialysis membrane (MW: 10.0 kDa) to eliminate the ammonium sulfate [24]. Eventually, the concentrated solution was dissolved in KPB buffer (0.05 M; pH 7.0) and loaded successively onto Q-sepharose columns with dimensions of 2.5 cm × 15 cm, which were first equilibrated with 50.0 mL KPB (0.01 M; pH 8.5) [25, 26]. The ChOx enzyme was purified by the elution buffer (0.05 M KPB buffer with 0.5 M NaCl) into different fractions at a flow rate of 10.0 mL per 1 h. The fractions (each containing 3.0 mL) of the target enzyme were merged after evaluating the enzymatic activity and protein content [11].

### Characterization and activity assay of ChOx enzyme

The molecular weight and purity of the ChOx enzyme were estimated via sodium dodecyl sulfate–polyacrylamide gel electrophoresis (SDS-PAGE) utilizing 12.5% acrylamide resolving gel and 4.5% acrylamide stacking gel with 0.1% SDS [25]. Protein samples were pre-treated with reducing buffer and heat-shocked at 90 °C for 5 min in a water bath. Specific enzyme activity was used to ensure that approximately equal amounts of the target enzyme were loaded into the gel. For this purpose, each sample was diluted to reach the specific enzyme activity of 0.05 U/mg. Then, 20.0 μL of each sample was loaded into individual lanes of the polyacrylamide gel along with the molecular weight standard marker (10.0 to 240 kDa). After two consecutive separation times (30 V for 40 min and 70 V for 120 min) were completed, the obtained protein bands were stained with Coomassie brilliant blue R-250 [18].

The catalytic activity of ChOx was measured by monitoring the production of quinoneimine dye at 500 nm [15]. For the assay, the reaction mixture was prepared with 2.5 mM cholesterol dissolved in 1.0% Triton X-100, 0.2 mg/mL ChOx solution, 1.0 µM of 4-AMAP, 15.0 µM phenol, and 10.0 U/mL HRP in 0.1 M KPB (pH 7.0). This mixture was incubated at 37 °C for 2 min. To determine the best cholesterol solvent, the enzyme activity was tested with three different detergents (Tween 20, Tween 80, and Triton X-100). Additionally, several physiochemical factors were evaluated to optimize the purified enzyme activity from *E. fergusonii*. These factors included various pH levels (5.0–10.0), temperatures (15–60 °C), and different storage times (2–100 days), and the effect of some metal ions (Ca^2+^, Mg^2+^, Cu^2+^, K^+^, Zn^2+^, Cd^2+^, Fe^2+^, and Mn^2+^) were estimated in KPB with a final reaction volume of 2.0 mL through quantifying the enzyme activity at each condition.

The thermostability analysis of the ChOx was carried out according to the previously reported method [27]. Briefly, a constant amount (0.2 mg/mL) of the enzyme was incubated at four different temperatures (35, 45, 55, and 65 °C). During the heat treatment, the samples were drawn at 15-min intervals, and the residual activity (*A*_*Res*_) was measured after cooling the samples on ice. The melting temperature (*T*_*m*_) value of the enzyme was obtained according to the plot of *A*_*min*_/*A*_*0*_ (*A*_*min*_ is the minimum level of activity and *A*_*0*_ is the initial activity of the enzyme) against temperature (K). Thermodynamic parameters, including enthalpy (*ΔH*^*#*^_*IN*_), Gibs free energy (*ΔG*^*#*^_*IN*_), and entropy (*ΔS*^*#*^_*IN*_), were calculated according to the following equations (Eq. 1–3) [28]:

1 $${\Delta H}_{IN}^{\#}={E}_{a}^{\#}-RT$$

2 $${\Delta G}_{IN}^{\#}=-RT\times \text{ln}(\frac{{k}_{IN}\times h}{{K}_{B}\times T})$$

3 $${\Delta S}_{IN}^{\#}=({\Delta H}_{IN}^{\#}-{\Delta G}_{IN}^{*})/T$$

where *E*^*#*^_*a*_, *R*, *T*, and *k*_*IN*_ depict the activation energy for the thermal inactivation of the enzyme, universal gas constant (8.314 J/mol.K), temperature (K), and first-order rate of thermal inactivation of the enzyme activity, respectively. In addition, *h* and *k*_*B*_ correspond to Planck’s constant and Boltzmann constant, which were 6.63 × 10^–34^ J.s and 1.38 × 10^–23^ J/K, respectively.

Furthermore, the following equation (Eq. 4) was applied to obtain the half-life (*t*_*1/2*_) of the extracted ChOx at the respective temperatures [28].

4 $${t}_{{}^{1}\!\left/ \!{}_{2}\right.}=ln\frac{2}{{k}_{IN}}=\frac{0.693}{{k}_{IN}}$$

The enzyme-specific activity was measured through the cholesterol (0–2.4 mM) standard curve and reported in units of [U/min] under the optimum condition of the experiment with a repetition number of 3 times [29, 30]. As essential parameters for evaluating enzyme function, the kinetic parameters of optimized ChOx, including the values of maximum velocity (*V*_*max*_) and Michaelis-Menthen constant (*K*_*m*_), were investigated with the help of a Lineweaver–Burk plot. For this goal, a diverse range of cholesterol (0.1–3.0 mM) was added to the reaction mixture in the optimized condition, and the absorption of the quinoneimine dye as the final product was investigated at 500 nm. The accurate kinetic parameters of catalase were defined according to Lineweaver–Burk equation (Eq. 5) [31]:

5 $$\frac{1}{{V}_{max}}= \frac{{K}_{m}}{{V}_{max}} . \frac{1}{[S]}+ \frac{1}{{V}_{max}}$$

where *[S]* refers to the substrate concentration.

### Artificial neural network (ANN) modeling

The growth of a microorganism is naturally affected by biological diversity [32]. In the present study, the ANN approach was tested to model *E. fergusonii* growth for higher ChOx production and compare the network model prediction against experimental results. Data was collected by screening a growth experiment in 10.0 mL media in 25.0 mL Erlenmeyer flasks. The flasks were incubated at different combinations of temperatures, pH, incubation time, cholesterol concentration, shaking speed, and various concentrations of MgSO_4_, NaCl, (NH_4_)_2_SO_4_, and K_2_HPO_4_ in a shaker incubator. After bacterial growth, the efficiency of enzyme production was studied by evaluating the cholesterol oxidase enzyme catalytic activity using the Sasaki method. On the other hand, the impact of different kinds of variables, including temperatures, pH, storage time, and organic salts (including MgSO_4_, KCl, CuSO_4_, CaCl_2_, MnCl_2_, CdCl_2_, FeCl_2_, and ZnSO_4_) on the catalytic activity of the extracted enzyme was tested. For this purpose, a multilayer feed-forward back propagation perceptron was created using Matlab 8.2 to model the possible effects of different factors on enzyme production through the fermentation process as well as the catalytic activity of the extracted enzyme. The ranges of investigated variables are shown in Table 1.

**Table 1:** The ranges of different input and output variables in ANN modeling for fermentation process and enzyme stability

Fermentation	Variable (Input layer)	pH	[Cholesterol] (g/L)	Temperature (◦C)	Shaking speed (rpm)	Incubation time (h)	[Metal salts] (g/L)
	Range	5.0–10.0	0.05–2.0	15–65	50–250	12–132	0–0.5
Activity	Variable (Input layer)	pH	Temperature (◦C)	Storage time (days)	[Metal ions] (ppm)	-	-
	Range	5.0–10.0	15.0–65.0	1–80	0–200	-	-

The developed ANNs were composed of the input layer represented by 9 (the fermentation process) and 11 (for extracted enzyme activity) independent factors, the output layer with only one neuron (SA%), and 8 and 7 neurons in the hidden layer, respectively. ANNs contained nodes with the hyperbolic tangent sigmoid (tansig) activation function (Eq. 6) for the hidden layers and a linear function (purelin) (Eq. 7) for the output layers. The error function minimization was performed using the Levenberg-Marquart (trainlm) algorithm. The data used for developing ANNs was normalized in the range of -1 to + 1 using Eq. 8 (Eq. 8) and divided into three different data sets, including training, validation, and test data. The mean squared errors (MSE) were calculated to obtain the number of neurons in the hidden layer and create the best ANN model design [33, 34].

6 $$Tansig(x)=\frac{2}{1+\mathit{exp}(-2x)}-1$$

7 $$Purelin(x)=x$$

8 $${Y}_{norm}=2(\frac{{Y}_{i}-{Y}_{i min}}{{Yi{,}min}_{i{,}max})-1}$$

where *Y*_*i, min,*_ and *Y*_*i, max*_ are the lowest and highest values of the variable *Y*_*i*_, respectively.

## Results and discussion

### Screening of microorganisms

Almost sixty bacterial single colonies were isolated from the total 15 whey samples in the initial screening based on their ability to grow on LB as the isolation medium; subsequently, each single colony was probed for extracellular ChOx production using an initial screening medium containing cholesterol as the sole carbon source. Indeed, ChOx catalysis is the oxidation of cholesterol with the synchronous production of cholest-4-en-3-one and H_2_O_2_ as products. The formation of red halos around the colonies illustrated the existence of ChOx activity, which is attributable to the formation of quinone imine dye in the presence of cholest-4-en-3-one, 4-AMAP, phenol, and HRP enzyme [11]. Among them, 9 isolates (A5, A6, A11, B7, B15, C2, C8, C13, and C19) revealed noticeable extracellular ChOx activities in qualitative detection by allowing the bacterial cultures to grow on the ChOx indicator plate. In order to investigate the selected isolates, they were cultured in cholesterol-enriched medium broth as an enzyme source. Then, the cholesterol degradation capability of the produced enzymes was investigated by monitoring the absorbance of the medium at 240 nm and 500 nm, which are assigned to cholest-4-en-3-one and quinoneimine, respectively. Out of the 9 positive isolates, a strain (B7) with relatively higher ChOx-specific activity of 1.02 U/mg compared to the other eight bacterial isolates (Table 2) was selected for further optimization studies and enzyme production.

**Table 2:** Screening of selected isolates for extracellular ChOx-specific activity

Strain number	Name	Red-colored colonies	ChOx (U/mg)
1	A5	+	0.54
2	A6	+	0.85
3	A11	+	0.25
**4**	**B7**	** + **	**1.02**
5	B15	+	0.72
6	C2	+	0.89
7	C8	+	0.67
8	C13	+	0.23
9	C19	+	0.61

### Identification and characterization of target isolate

**Fig. 1:**
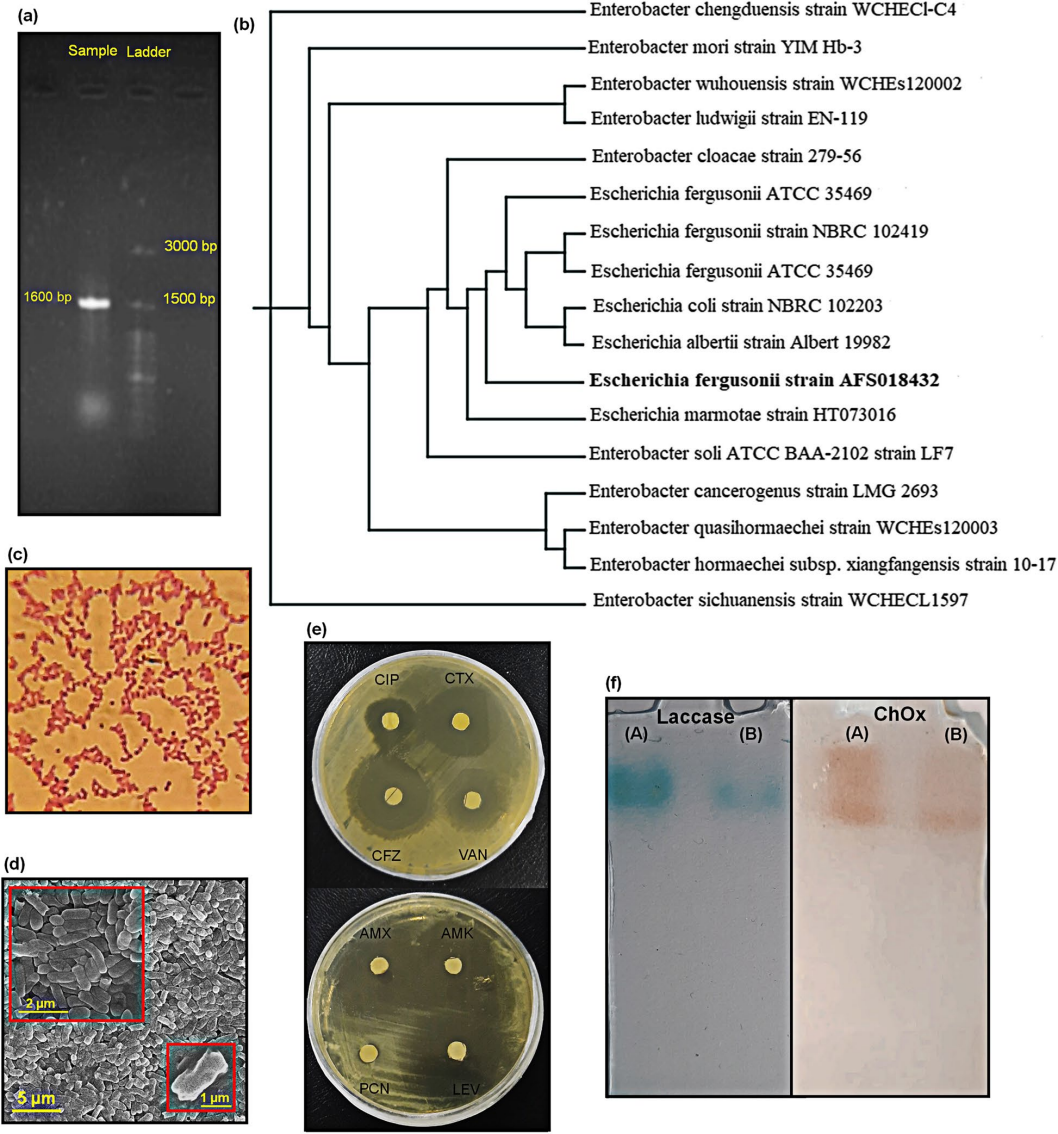
Agarose gel electrophoresis of 16S rRNA PCR product (**a**), phylogenetic tree based on 16S rRNA sequence and the closest relatives (**b**), gram staining result (**c**), SEM images (**d**), and antibiogram (**e**) of the *E. fergusonii*. strain (OQ257372.1), and zymogram patterns of laccase and ChOx for the crude enzyme solution (A) and its 1:4 dilution (B) (**f**)

The taxonomic identification of the target strain was performed based on partial nucleotide homology and phylogenetic analysis. The amplified 16S rRNA of the ChOx-producing strain using universal primers was analyzed by 0.8% gel electrophoresis (Fig. 1a) and then sequenced and compared with other 16S rRNA gene sequences available in the Gen Bank database of NCBI using BLAST software [19]. The 16S rRNA gene sequence of the newly isolated strain showed the highest similarity to *E. fergusonii*, with 98.96% identity. This sequence has been deposited in the NCBI GenBank under the accession number OQ257372.1. Furthermore, a phylogenetic tree was constructed involving the closest relatives based on molecular taxonomy. As depicted in Fig. 1b, the target strain exhibited a maximum sequence homology of 94.16% and 98.95% with many *Escherichia* sp. Therefore, the strain was also confirmed as *E. fergusonii*, a gram-negative and rod-shaped species (Figs. 1c and d). The antibiotic sensitivity pattern of the target isolate illustrated the most resistance to PCN (92.5%) and the best sensitivity to AMX (52.5%), among others. From the results of the antibiogram (Fig. 1e), the *E. fergusonii* isolates in this study revealed 82.5%, 75.0%, 70.0%, 62.5%, 60.0%, and 49.0% resistance against CIP, VAN, CTX, CFZ, AMK, and LEV, respectively. This study used the zymography approach, a technique that analyzes enzymatic systems through a staining process, to determine other oxidase enzymes produced by *E. fergusonii* As illustrated in Fig. 1f, the case-studied strain illustrated no glucose oxidase or peroxidase activity, which implies the specific metabolic pathways present in this strain. However, it can secrete laccase along with ChOx. The obtained zymogram patterns illustrated stronger bands for the crude samples and fainter bands for the 1:4 dilutions.

### Optimization of fermentation condition for maximum ChOx production

**Fig. 2:**
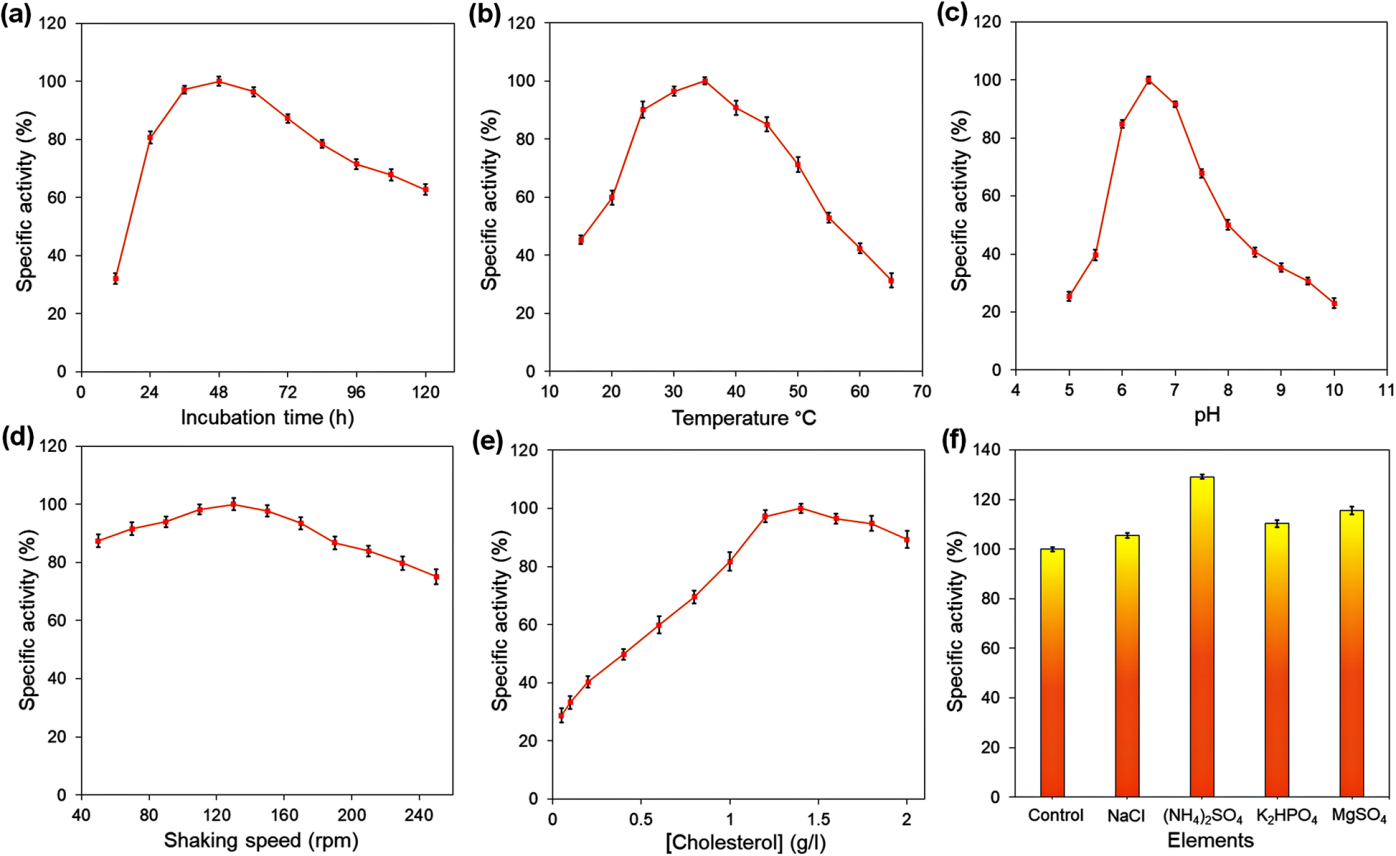
Effects of different parameters, including incubation periods (**a**), temperatures (**b**), pH value (**c**), shaking speed (**d**), cholesterol concentration (**e**), and some nutrient elements (**f**) on the cholesterol decomposition by *E. fergusonii*

The effects of different parameters on the fermentation process were investigated using the one variable at a time (OVAT) approach to achieve the maximum cholesterol oxidase production. Indeed, the ChOx activity was considered an enzyme production index and ranged from 0.007 to 1.09 U/mg. For this purpose, duplicate static conditions were applied throughout the fermentation study with a final reaction volume of 5.0 mL in KPB (0.1 M; pH 7.2) containing 0.1% cholesterol and an equal bacterial unit (0.5 × 10^6^ CFU/mL) and incubated at 37 °C for 36 h. As illustrated in Fig. 2a and b, the best incubation time and temperature for extracellular ChOx produced by the target strain were 48 h and 35 °C, respectively. The effect of different pH values ranging from 5 to 10 revealed the highest ChOx production at pH 6.5 (Fig. 2c). Additionally, the results displayed the highest cholesterol decomposition property at a speed of 130 rpm and in the presence of 1.4 g/L cholesterol (Fig. 2d and e). The effect of some significant variables, namely NaCl, (NH_4_)_2_SO_4_, K_2_HPO_4_, and MgSO_4_, was investigated with a final concentration of 0.5 g/L. The obtained data illustrated that the presence of the mentioned compounds (especially (NH_4_)_2_SO_4_) remarkably increased ChOx production (Fig. 2f).

### Isolation and purification of ChOx

The crude enzyme was isolated from the supernatant through cold centrifugation of the bacterial growth media, which was subsequently precipitated by ammonium sulfate salt up to 60.0% saturation and dialyzed utilizing KPB (0.02 M; pH 7.0) for the partial purification of the enzyme. Applying this condition led to the fraction obtained with ammonium sulfate illustrating an increase in specific activity from 1.14 U/mg protein for the raw enzyme to 6.11 U/mg protein, which relates to 5.35-fold purification. In the next step, further purification of the prepared solution was obtained by loading the preparation into a Q-Sepharose gel chromatography column. The initial adjusted pH of 8.5 made the target fractions containing ChOx collect in the flow-through fraction. In contrast, the second Q-Sepharose column with a pH value of 10.0 led to the adsorption of ChOx to the column, which was washed with elution buffer with 0.5 M NaCl. Finally, active parts of the fractional purification were gathered, pooled, and concentrated by dialysis against the same buffer [12]. The chromatographic phase resulted in 13.86‐fold purification of the enzyme with a protein content of 93.0 mg/mL and a specific activity of 15.8 U/mg (Table 3) [3].

**Table 3:** Summary of purification phases of the ChOx produced by *E. fergusonii*

Purification phases	Total activity (U/mL)	Protein content (mg/mL)	Specific activity (U/mg)	Purification folds		Yield (%)
Cell-free filtrate (crude)	4827	4235	1.14	1.00	100	
Saturation	3617	592	6.11	5.35	74.9	
Chromatography	1287	93.0	15.8	13.86	26.6	

### Some properties of isolated ChOx

**Fig. 3:**
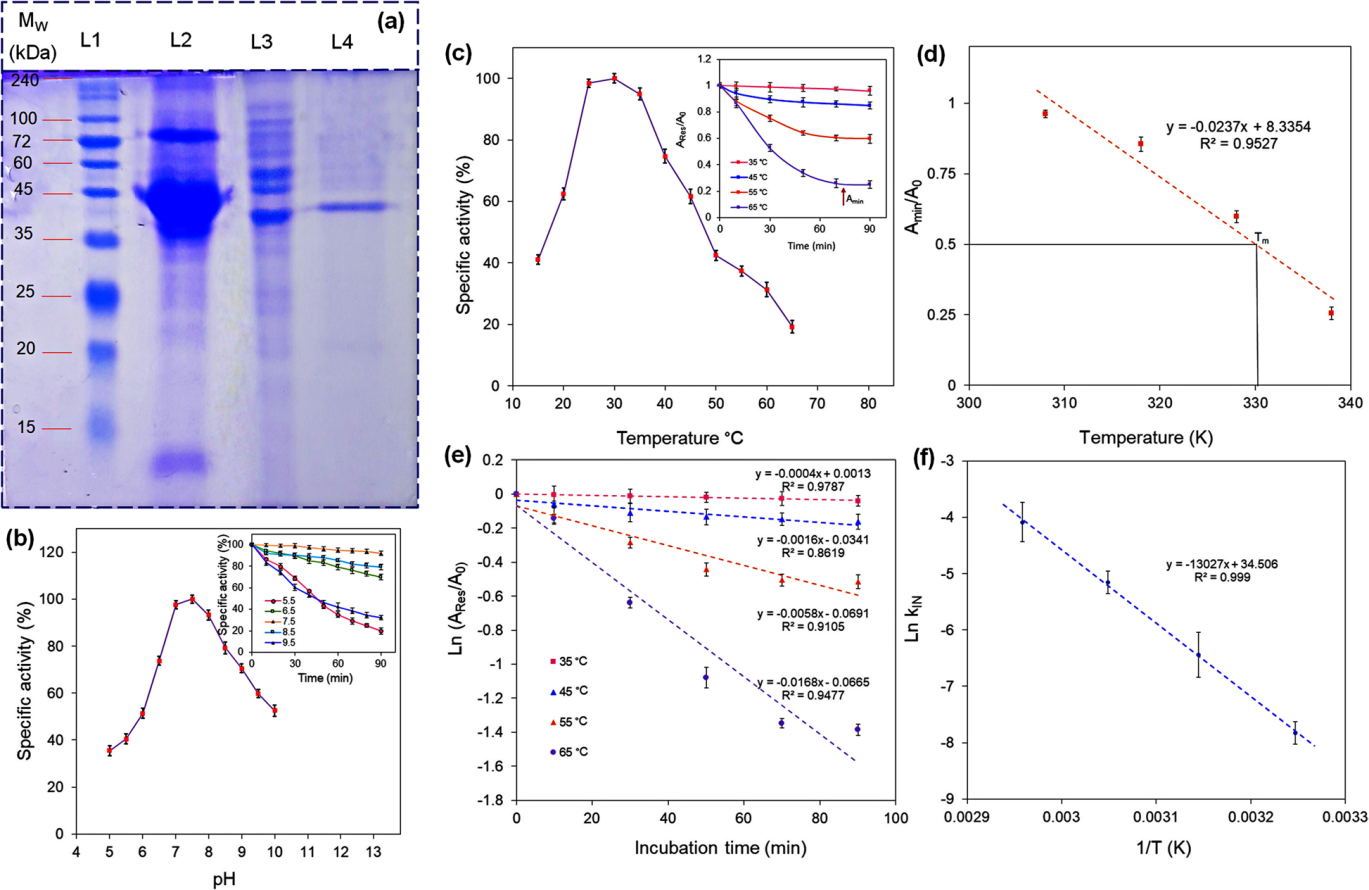
SDS-PAGE electropherogram of purification steps: SDS-PAGE analysis L1: molecular weight markers, L2: crude enzyme, L3: ammonium sulfate precipitated enzyme, and L4: indicating ChOx enzyme purified by Q-sepharose column (**a**), effects of different pH value on the ChOx activity (inset: pH stability) (**b**), the effects of different temperatures on the ChOx activity (inset: reduction in the proportion of *A*_*Res*_ compared with *A*_*0*_ over time( (**c**), estimation of melting temperature *T*_*m*_ (**d**), first order plot to calculate the rates of thermal inactivation (*k*_*IN*_; 1/s) (**e**), and Arrhenius plot (the slope gives the activation energy of denaturation, *E*.^*#*^_*a*_ (J/mol)) (**f**)

Homogeneity analysis of the purified enzyme, which was carried out by 12.5% SDS-PAGE, illustrated the successful purification of the enzyme with a single protein band. As shown in Fig. 3a, the molecular weight of the pure ChOx was estimated to be 49.0 kDa by the characteristics of the electrophoretic pattern. The effects of different parameters on enzymatic activity were investigated in KPB (0.1 M; pH 7.0) with a final volume of 2.0 mL at 37 °C. Exposure of ChOx to different detergents (Tween 20, Tween 80, and Triton X-100) with a final concentration of 0.1% (v/v) for 60 min led to retaining the enzyme activity up to 58.0%, 74.0%, and 87.0%, respectively. However, the presence of SDS resulted in a complete inhibition of the enzyme, so Triton X-100 was selected for further analysis. The newly isolated ChOx enzyme exhibited sufficient activity in the pH range of 5.0 to 10.0 (Fig. 3b), which allows it to work efficiently over a wide range of pHs. Nevertheless, the maximum activity was obtained at pH 7.5. This enzyme was able to preserve about 75.0% of its initial activity at pH values ranging from 6.5 to 8.5. Furthermore, the effect of different pH on the ChOx stability was evaluated by incubating the enzyme under different conditions for 90 min (Fig. 3b, insets). The thermal stability of the purified enzyme was accomplished at different temperatures ranging from 15 to 65 °C. As demonstrated in Fig. 3c, the ChOx enzyme activity reached the maximum rate of reaction at 30 °C, and thereafter, increasing temperature resulted in a gradual decrease in enzyme activity. Despite the alteration in ChOx enzymatic function, it maintained almost 28.0% of optimal activity at high temperatures of 65 °C, respectively. The thermostability of an enzyme refers to its resistance to attain irreversibly denatured form [35]. The remaining activity of the extracted ChOx after heat treatment at 35, 45, 55, and 65 °C was measured in the presence of cholesterol (as a substrate). As shown in Fig. 3c, insert, a remarkable reduction in the activity of ChOx was observed with increasing temperature, and a constant residual activity was observed after 90 min of incubation. The *T*_*m*_ is one of the most important thermostability parameters, which was calculated using Fig. 3d to be 57.53 °C. Tm is considered the temperature when *A*_*min*_ decreases to 50.0% of *A*_*0*_. The *k*_*IN*_ values, first-order rate constants of inactivation [24], were obtained according to Fig. 3e (slope = *k*_*IN*_), which was then applied for the calculation of the activation energy of enzyme inactivation (*E*^*#*^_*a*_) using the Arrhenius plot (slope = *-E*^*#*^_*a*_*/R*) [24] to be 108.306 kJ/mol (Fig. 3f). The half-life of the enzyme decreased as the temperature increased (Table 4). Thermodynamic parameters were calculated, and the results are listed in Table 4.

**Table 4:** The calculated thermodynamic parameters of the activation of enzyme inactivation at various temperatures

T (K)	ΔS^#^_IN_(J/mol.K)	ΔH^#^_IN_(KJ/mol.K)	ΔG^#^_IN_(KJ/mol.K)	T_1/2_(min)
308	33.0	105.74	95.55	1732.5
318	33.3	105.66	95.07	433.12
328	33.4	105.57	94.63	119.48
338	32.1	105.49	94.61	41.25

Additionally, the enzyme was almost stable for a storage period ranging from 2 to 80 days at 4 °C (Table 5). As the stability profile shows, the ChOx enzyme retained 72.0% of its activity during the experiment. The ChOx enzyme was individually exposed to some metal ions with a final concentration of 200 ppm for 60 min, and then the activity was examined (Table 5). As a result, just Mg^2+^ increased the enzymatic activity by 24.0%. Other metal ions, including Cu^2+^, K^+^, Fe^2+^, Zn^2+^, Mn^2+^, Ca^2+^, and Cd^2+^, remarkably inhibited the enzymatic activity, ranging from 20.0 to 55.0% in comparison with the initial velocity.

**Table 5:** The effects of metal ions on the ChOx activity

Metal ions (200 ppm)	Relative activity of ChOx (%) ± SD	Storage period (days)	Relative activity (%) ± SD
None	100	1	100 ± 0.8
Mg^2+^	124 ± 1.8	10	99.0 ± 1.2
Cu^2+^	79.0 ± 1.5	20	97.5 ± 1.4
K^+^	96.5 ± 1.2	30	95.1 ± 1.2
Fe^2+^	80.0 ± 1.3	40	91.3 ± 0.9
Zn^2+^	81.0 ± 1.9	50	82.0 ± 1.0
Mn^2+^	67.0 ± 1.2	60	78.0 ± 0.9
Ca^2+^	87.0 ± 1.1	70	77.0 ± 0.5
Cd^2+^	45.0 ± 2.4	80	72.0 ± 1.8

In order to determine the kinetic parameters (*K*_*m*_ and *V*_*max*_) of the isolated ChOx enzyme from *E. fergusonii*, the linear regression of Lineweaver–Burk plots [36] was utilized under optimum conditions. The results illustrated that enzyme activity was enhanced linearly with increasing the cholesterol concentration, from 0.1 to 2.4 mM. However, higher concentrations of cholesterol lead to a deviation from the linear mode. Accordingly, the values of *K*_*m*_ and *V*_*max*_ were estimated to be 0.71 mM and 21.7 U/mL, respectively [13].

### ANN analysis results

**Fig. 4:**
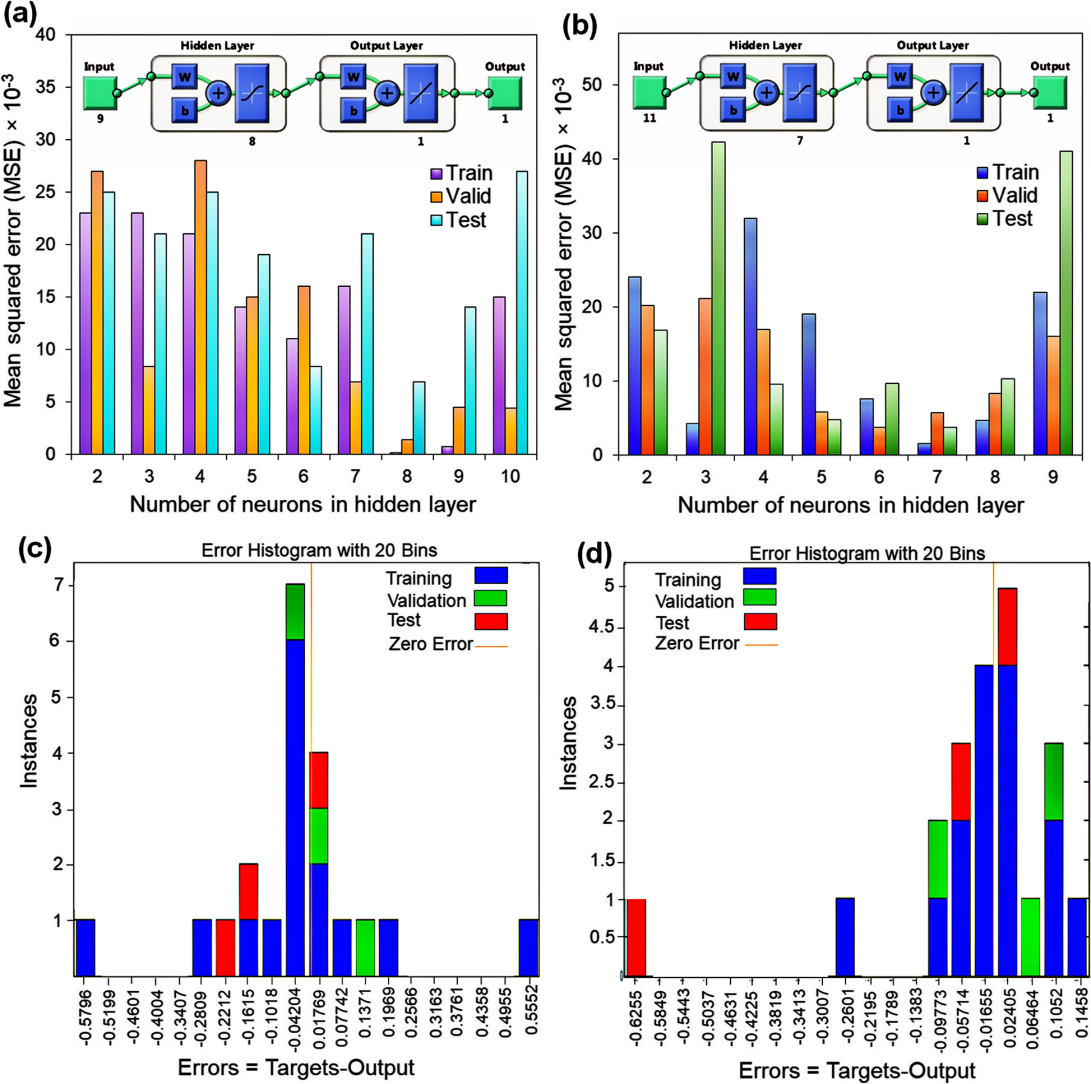
Optimization of the number of neurons in the hidden layer for ChOx enzyme production (**a**), and extracted ChOx activity (**b**) (insets are the best corresponding topology design), the error histograms of all data for ChOx production (**c**), and ChOx catalytic activity (**d**)

**Fig. 5:**
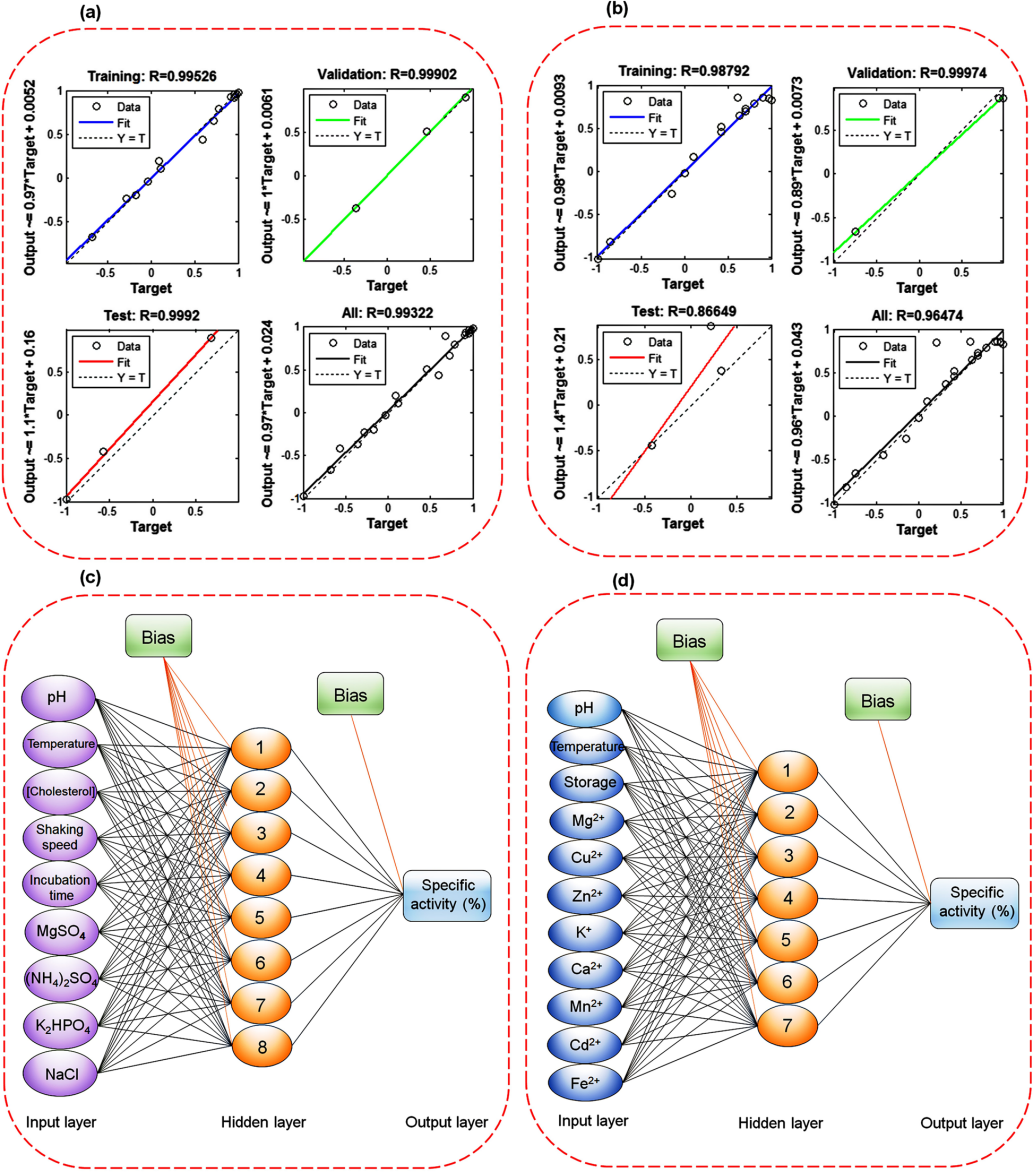
The plots of the experimental data against the ANNs predicted results for training data, validation data, test data, and all data; fermentation process for enzyme production (**a**), extracted enzyme activity (**b**), schematic illustration of the optimized ANNs with 9:8:1 and 11:7:1 topologies for fermentation process for enzyme production (**c**), and extracted ChOx activity (**d**)

**Fig. 6:**
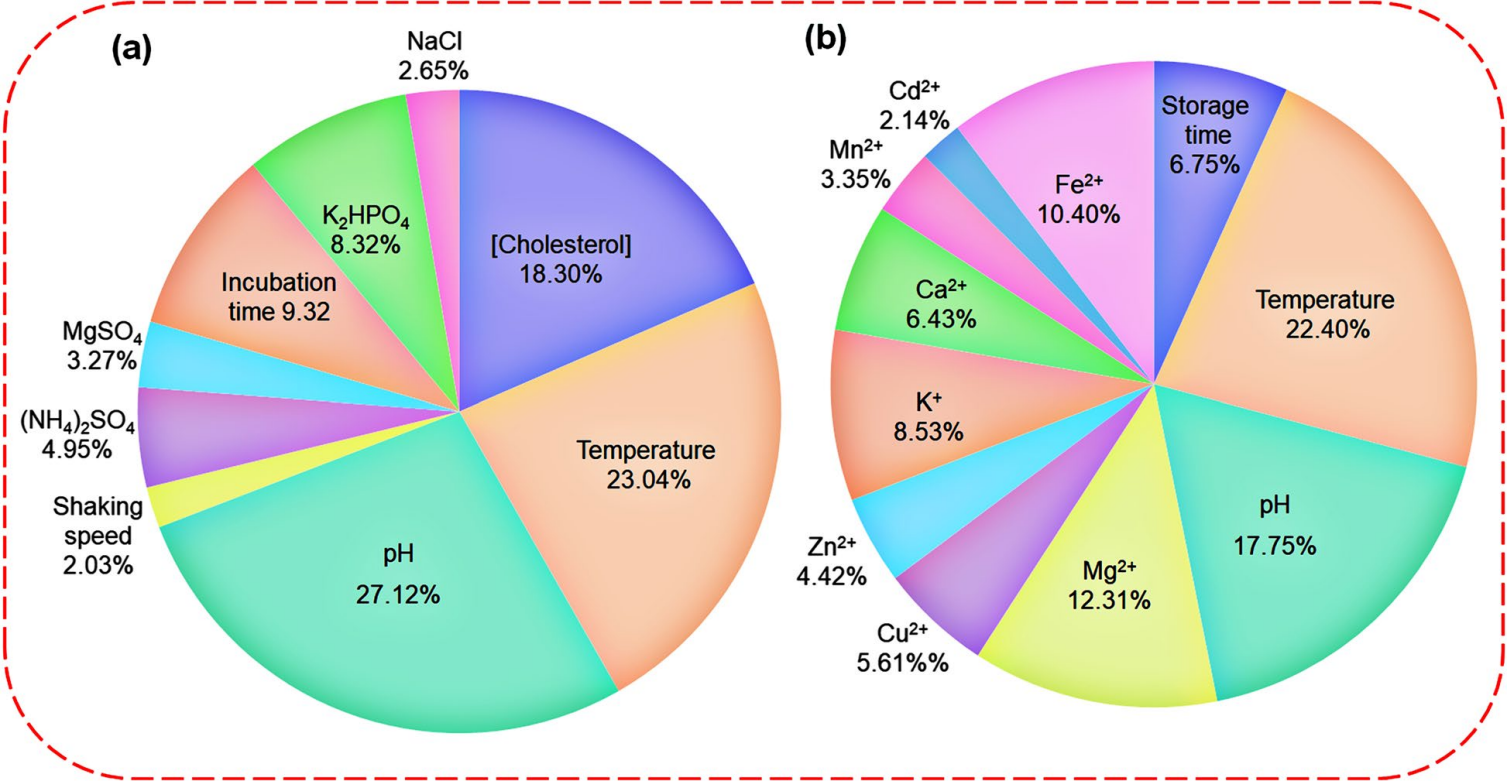
The possible effects of different input variables on the ChOx enzyme production (**a**) and enzyme activity (**b**)

ANNs are information processing systems that constitute interconnected processors called neurons that resemble the human nervous system and can model complex patterns and make predictions [37]. The ANN approach was found to be widely applied in the optimization of fermentation processes [38]. The data obtained from the experimental design was applied to determine the optimal architecture of ANNs. The results are presented in Fig. 4. As shown in Figs. 4a and b, the hidden layers with 8 and 7 nodes had the lowest value of MSE for optimizing the fermentation process and the assessment of the catalytic activity of the enzyme, respectively. Therefore, in the present study, three feed-forward back propagation perceptron ANNs with 9:8:1 and 11:7:1 topologies were developed to model the bacterial growth and the isolated enzyme activity in the presence of different variables. The error histograms were plotted, and the findings indicated that most of the data were scattered around the zero line (Fig. 4c and d). In addition, Fig. 5 a and b depict the plots of Ann’s predicted results against experimental data. According to these figures, all the data were excellently distributed around the line y = x. Therefore, based on the good correlations between training and validation data, it can be concluded that the designed ANNs had good abilities to predict the data and respond to unexpected conditions. On the other hand, the high R-value of the test data further indicated that the developed architectures were not overfitting the training data [33, 39]. Figure 5c and d represent the schematic illustration of the optimized ANN structure. Also, the outcomes revealed that pH and temperature had the most significant influence, with a relative relevance of 27.12% and 22.4%, on the fermentation process and enzyme activity, respectively (Fig. 6 a and b).

## Discussion

As the enzyme market grows, understanding the capacity of bacteria to produce enzymes becomes crucial for discovering commercially valuable enzymes. Various Gram-positive and Gram-negative bacteria members of the genera *Chromobacterium* [40, 41], *Brevibacterium *[42], *Pseudomonas* [43], *Mycobacterium* [44], *Nocardia* [45], *Rhodococcus* [46], *Streptomyces* [11], and *Chromobacterium* [47] from varied soil and food sources are reported to produce ChOx with distinct characteristics. However, most of them do not apply to the industrial-scale bioproduction of the ChOx enzyme. In this work, a total of 9 colonies that were able to grow in cholesterol enrichment medium were isolated from local whey samples. Among them, the most effective strain for cholesterol degradation was identified through molecular analysis as *E. fergusonii*. To the best of our knowledge, no research has introduced *E. fergusonii* as a ChOx producer. The antibiotic sensitivity pattern of E. fergusonii illustrated the most resistance to PCN (92.5%) and the best sensitivity to AMX (52.5%). These measurements were then compared to CLSI (Clinical & Laboratory Standards Institute) standards to determine whether the bacteria were susceptible, intermediate, or resistant to the antibiotics tested. In industrial and research processes, it is crucial to examine the antibiotic resistance profile of the bacterial strain to manage the contamination risks in the enzyme production process. To improve the productivity of ChOx by *E. fergusonii*, the influence of some affecting elements (incubation time, temperature, pH, cholesterol concentrations, shaking speed, and various metal ions) on the growth and decomposition of cholesterol in broth media was evaluated. The variation in enzyme activity under different fermentation conditions highlights the importance of optimizing growth conditions and nutrient factors for enzyme production [24]. In the case of incubation time, the optimal result for enzyme activity was obtained after 48 h, which is different from other strains reported in the literature [48, 49]. This variation could be attributed to differences in metabolic rates or enzyme stability across different bacterial species. Additionally, increasing the incubation temperature from 15 to 35 °C improved ChOx production, but higher temperatures resulted in reduced enzyme activity, likely due to the thermal denaturation or instability of the enzyme, which is consistent with similar optimization studies for ChOx production from *Bacillus subtilis* [50]. In accordance with previous studies on various strains, our findings indicated that the optimal pH for ChOx production is around 6.5 [49, 51]. The concentration of cholesterol also significantly influenced bacterial growth and enzyme production. Our results showed that a cholesterol concentration of 1.4 g/L was optimal for ChOx production. This higher cholesterol concentration required for maximum enzyme activity revealed that the case-studied strain may have an enhanced capacity for cholesterol decomposition compared to other strains [48]. Furthermore, the presence of mineral salts notably increased ChOx production, with the most influence from (NH_4_)_2_SO_4_. This finding is consistent with the role of mineral salts in enhancing enzyme production observed in studies of chitinase from *E. fergusonii*. The enhanced enzymatic activity in the presence of these salts can be attributed to improved bacterial growth, which in turn supports higher enzyme yields [9]. These results emphasize the need for precise control of fermentation conditions to maximize enzyme yields. This suggests that other oxidase enzymes are alongside ChOx during bacterial metabolism. In addition to ChOx, various oxidase enzymes may also be produced during bacterial metabolism [21, 22]. The zymogram test for laccase activity resulted in a blue coloration when TMB was used as the substrate. This color change occurs because laccase, an oxidase enzyme, catalyzes the oxidation of TMB in the presence of molecular oxygen, producing a blue-colored oxidized form of TMB. This reaction is indicative of the presence of laccase activity within the sample. The absence of colored bands in the zymogram for peroxidase and glucose oxidase, despite using TMB as a substrate, suggests that these enzymes were either not produced by *E. fergusonii* or were present in quantities too low to be detected under the test conditions. Unlike laccase, which can directly oxidize TMB in the presence of molecular oxygen, peroxidase requires hydrogen peroxide as a co-substrate to catalyze the oxidation of TMB, leading to a color change. Similarly, glucose oxidase oxidizes glucose to produce hydrogen peroxide, which then reacts with TMB in the presence of peroxidase [22, 23].

The purified ChOx enzyme represented a single band in the SDS-PAGE pattern with a molecular weight of 49 kDa. This result is in good agreement with previous studies that have reported molecular weights for ChOx ranging from 30.0 to 61.0 kDa. The consistency of this result with existing data supports the reliability of our enzyme purification process [13, 42]. Enzyme behavior under specific conditions is crucial for its application in various fields, making it important to identify the optimal conditions for ChOx activity. In our study, we used the OVAT approach to investigate the effects of different factors on enzyme activity. Among the tested detergents, Triton X-100 yielded the highest enzyme activity. This stability of ChOx in the presence of Triton X-100 is in agreement with previous reports [12, 13, 26]. Similarly, Rodrigues et al. [25] highlighted the stability of ChOx from *Enterobacter cloacae* in the presence of detergents, suggesting that detergent stability is a common feature of this enzyme. In terms of pH, the ChOx enzyme from our isolated strain exhibited the highest activity at pH 7.5, which is in accordance with the findings of Cheetham et al. [45] for ChOx from *Nocardia sp.* and *Rhodococcus sp.* Meantime, most previous works estimated an approximately similar optimum pH for ChOx received from *Bacillus* sp. [13], *Brevibacterium sterolicum* [52], *Streptomyces violascens* [53], *Brevibacterium sterolicum*, *Nocardia rhodochrous* [45], and *Pseudomonas* sp. [54]. However, ChOx isolated from *Castellaniella* sp. [55] has been reported to function optimally at a pH of 8.0 [56]. Our study also found that the ChOx enzyme displayed strong stability against a range of pH levels, which is in agreement with previous research on ChOx stability [12, 41, 57, 58].

Temperature also plays a critical role in the cholesterol degradation capabilities of microorganisms. In our study, the maximum cholesterol oxidation by the target strain occurred at 30 °C, which is consistent with the findings of Ghaly et al. [18] for ChOx from *L. plantarum.* While other studies have reported different optimal temperatures for the isolated ChOx enzyme from *E. hirae* [3], *Enterobacter* sp. [26], *Brevibacterium sterolicum* [59], and *Streptomyces fradiae* [60] as 45–55 °C, 25 °C, 50 °C, and 70 °C, respectively. It seems that higher temperatures provide more kinetic energy to enzyme molecules, increasing the thermal denaturation of their three-dimensional structure, which is crucial for their catalytic activity [36, 37]. The rising temperature accelerates the rate of denaturation, which results in a shorter half-life. The higher value of *ΔG*^*#*^_*IN*_ reflects the more thermal stability of the enzyme. As well, there is a direct and indirect relationship between *ΔG*^*#*^_*IN*_ with *ΔH*^*#*^_*IN*_ and *ΔS*^*#*^_*IN*_, respectively [38]. *ΔH*^*#*^_*IN*_ and *ΔS*^*#*^_*IN*_ values decreased and increased, respectively, demonstrating a decrease in enzyme stability with increasing temperature [24, 39]. The positive values of *ΔS*^*#*^_*IN*_ and *ΔH*^*#*^_*IN*_ confirm the unfolding of the enzyme during heat treatment [39]. Based on the results obtained, the stability of the ChOx decreases with increasing temperature. However, the enzyme still retains a portion of its catalytic activity at elevated temperatures. For instance, after being incubated at 65°C for 90 min, the relative enzyme activity remains 28.0%. The enzymatic activity was further analyzed in the presence of various metal ions. Among all metal ions investigated in this work, just Mg^2+^ enhanced the enzyme activity, which was in agreement with some other bacterially isolated ChOx behaviors [3, 41, 55, 57]. The stimulatory effect of metal ions on ChOx activity can be attributed to their role in maintaining the enzyme’s structural integrity and facilitating electron transfer during the cholesterol oxidation process. Additionally, the activated enzymatic function may be assigned to the simulated coenzyme behavior of metals or the intermediacy function of the metal groups between the enzyme and substrate, thereby optimizing substrate binding and catalysis. In contrast, the binding of the metals to the active site might be responsible for the enzyme inhibition. Although some studies reported the positive effect of Cu^2+^ on the function of different ChOx enzymes [12, 18, 61], the enzyme obtained from *E. fergusonii* exhibited 27.0% inhibition under the effect of this metal ion. In some cases, the extracted ChOx activity was enhanced in the presence of Mn^2+^; however, this metal ion reduced the ChOx activity isolated from *E. fergusonii* in this work [54].

The kinetic parameters of enzymes, such as the *K*_*m*_ value, are crucial indicators of their catalytic efficiency and affinity for substrates. Various studies have reported a wide range of *K*_*m*_ values for the ChOx enzyme, reflecting differences in enzyme purity, source, and reaction conditions. For example, Km values of 0.12 mM, 0.15 mM, 0.16 mM, 3.25 mM, and 23.0 mM have been observed in *Enterobacter sp*. [26], *Streptomyces aegyptia* [11], *Castellaniella* sp [55], *Bacillus subtilis* [13], and *Brevibacterium* sp [41], respectively. Considering the results of the ANN analysis, the factors with the most positive impact on the production and activity of ChOx were determined to be pH and temperature. In the second grade, the concentration of cholesterol and Mg^2+^ efficiently enhanced ChOx production and activity, respectively.

## Conclusion

Herein, we identified a new bacterial strain from a local whey sample that has promising potential for producing maximum levels of ChOx to fulfill various requirements in different applications, including biosensor construction, steroid biotransformation, and biocontrol of insects. The obtained strain was identified at the morphological and molecular level based on the 16S rRNA gene region and deposited in the NCBI database as *E. fergusonii* with the accession number OQ257372.1. According to the literature, isolation of ChOx has not been reported from *E. fergusonii*, so this strain was introduced as a novel ChOx source. After optimization of growth conditions for abundant enzyme production, the purification step based on ammonium sulfate precipitation was followed by ion exchange chromatography utilizing the Q-Sepharose column to obtain the highest specific enzyme activity of 15.8 U/mg. The successful purification was verified using SDS-PAGE analysis by appearing as a single band for purified ChOx with a molecular weight of 49 kDa. Further biochemical characterization of the ChOx estimated the optimum condition for better enzymatic activity. Considering the beneficial properties, including thermal stability, pH tolerance, and endurance against detergents, the ChOx produced by E. fergusonii demonstrates promising characteristics for enzyme production. Additionally, AI modeling was employed to identify the key factors that influence maximum enzyme production and activity. Overall, this study provides fundamental information to develop successful, large-scale, and cost-effective production of extracellular ChOx for cholesterol monitoring.

## Supplementary Information

Supplementary Material 1.

Supplementary Material 2.

## Data Availability

https://www.ncbi.nlm.nih.gov/nuccore/OQ257372.1/.
