# Supplementary figures and images for: Optimized extraction and kinetic study of cholesterol oxidase from a newly isolated Escherichia fergusonii strain from local whey samples: insights through a combined experimental study and artificial neural network modeling
Source: BMC Microbiol. 2025 Jan 20;25:32. doi: 10.1186/s12866-024-03728-0 (PMC11744973; doi:10.1186/s12866-024-03728-0)

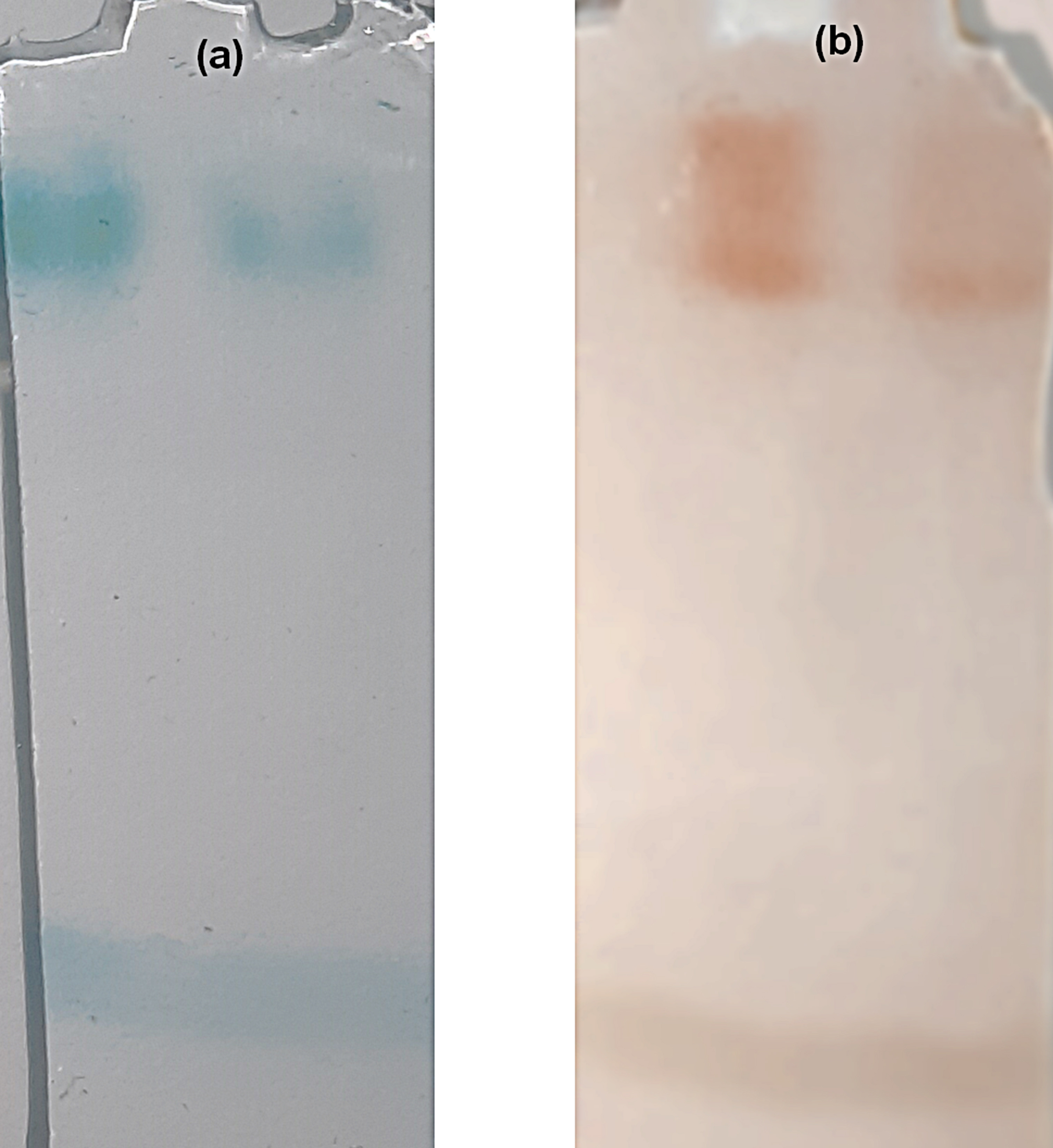

Supplement: Supplementary file 1 — Supplementary Material 1. [file 12866_2024_3728_MOESM1_ESM.jpg]

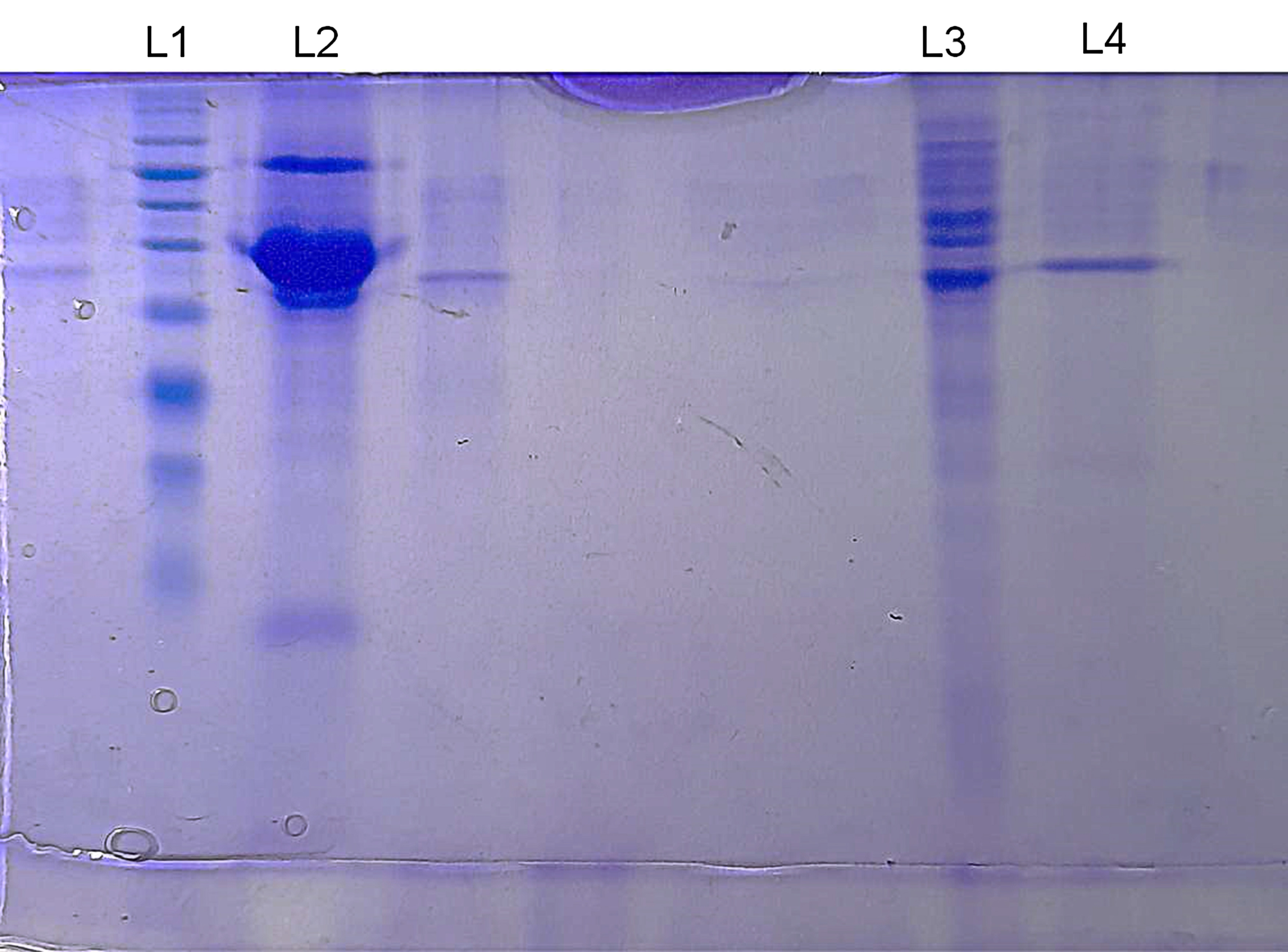

Supplement: Supplementary file 2 — Supplementary Material 2. [file 12866_2024_3728_MOESM2_ESM.jpg]
